# Sex Differences in Continuous Glucose Monitoring Metrics and Glucose Variability in Subjects with Type 1 Diabetes Treated with Advanced Hybrid Closed Loop Therapy: An Observational, Retrospective, One-Year Follow-Up Study

**DOI:** 10.3390/jcm14248823

**Published:** 2025-12-13

**Authors:** Matteo Conti, Ilaria Gironi, Elena Meneghini, Elena Mion, Giacoma Di Vieste, Federico Bertuzzi, Basilio Pintaudi

**Affiliations:** 1 Department of Medicine and Surgery, University of Milan-Bicocca, 20126 Milan, Italy; m.conti69@campus.unimib.it; 2 Diabetes Unit, Niguarda Cà Granda Hospital, 20162 Milan, Italy; ilaria.gironi@ospedaleniguarda.it (I.G.); elena.meneghini@ospedaleniguarda.it (E.M.); elena.mion@ospedaleniguarda.it (E.M.); giacoma.divieste@ospedaleniguarda.it (G.D.V.); federico.bertuzzi@ospedaleniguarda.it (F.B.)

**Keywords:** type 1 diabetes, advanced hybrid closed-loop, continuous glucose monitoring, sex difference

## Abstract

**Background**: Advanced hybrid closed-loop (aHCL) systems have improved glycemic control in individuals with type 1 diabetes (T1DM). However, it remains unclear whether their efficacy and safety differ by patient’s sex, in view of known sex-related physiological and behavioral differences in disease control and management. **Methods**: This retrospective, single-center study included 176 adults with T1DM starting aHCL therapy with Medtronic MiniMed™ 780G. Continuous glucose monitoring (CGM) metrics, glycated hemoglobin (HbA1c), and glycemic variability (GV) indexes were collected at baseline, 6 months, and 12 months after starting aHCL therapy. Only patients with at least 70% sensor usage were included at each time point. The primary outcome was the assessment of sex-related differences in CGM metrics at 12 months. Secondary outcomes included changes in HbA1c and GV indexes by sex and over time. **Results**: TIR increased significantly at 6 (+6.6%, *p* < 0.001) and 12 months (+5.4%, *p* < 0.001), TAR decreased, and TBR remained stable. HbA1c was significantly reduced at both 6 and 12 months (−0.6%, *p* < 0.001). Improvements were consistent in both males and females, with females exhibiting better improvement in HbA1c compared to males (−0.4%, *p* = 0.049). No significant sex differences were found in CGM metrics at 12 months. GV indexes improved significantly in both groups, regardless of sex. At the multivariable analysis, only HbA1c <7.0% at baseline was associated with the achievement of the composite outcome (TIR > 70%, TBR < 4%, HbA1c < 7.0%). **Conclusions**: aHCL therapy improved glycemic control and GV in adults with T1DM, regardless of the patient’s sex. These results support the generalizability of aHCL therapy and underscore the need to ensure equitable access to technologies rather than sex-specific adjustments.

## 1. Introduction

Type 1 diabetes mellitus (T1DM) is a chronic autoimmune disease characterized by absolute insulin deficiency, requiring insulin therapy to maintain glycemic control and prevent disease complications [1]. Advanced hybrid closed-loop (aHCL) systems have revolutionized diabetes management by automating insulin adjustments based on real-time glucose levels. This showed to improve metabolic control and a diminished burden of the disease for patients and caregivers, by optimizing glycated hemoglobin (HbA1c) levels, continuous glucose monitoring (CGM) derived metrics, and glycemic variability (GV) indexes [2,3]. Although these findings support the long-term benefits of aHCL therapy in subjects with T1DM, emerging evidence suggests that glycemic outcomes may be influenced by factors such as sex differences in insulin sensitivity, hormonal fluctuations, and behavioral aspects of diabetes management. Data from the T1D Exchange clinic registry showed that although women more frequently and carefully monitor their blood glucose, they often have higher HbA1c levels, suggesting that behavioral adherence alone does not fully explain sex differences in glycemic control [4]. Similarly, biological factors, such as sex hormones, differences in proinflammatory factors, and body fat distribution, have also been implicated in modulating insulin sensitivity and influencing the risk of microvascular and macrovascular complications [5]. Despite these known differences, evidence is still limited on whether aHCL systems perform equally across sexes, and understanding these differences is critical to achieve personalized diabetes care.

This study aims to evaluate potential sex-related differences in glycemic response to aHCL therapy in subjects with T1DM with a focus on CGM metrics, several glucose variability indexes, and glucose control over a maximum one-year follow-up period.

## 2. Methods

This was an observational, retrospective, single-center study. Consecutive subjects with T1DM being treated with the aHCL system Medtronic MiniMed^TM^ 780G (Medtronic MiniMed, Northridge, CA, USA) followed at Niguarda Hospital (Milan, Italy) were included. Inclusion criteria were as follows: age >18 years, T1DM, and first use of an aHCL system. Exclusion criteria were as follows: age <18 years, type 2 diabetes, gestational diabetes, pregnancy, and previous use of an aHCL system.

The study was performed in accordance with the Helsinki Declaration of 1964 and its later amendments and complied with national regulations. It was approved by the local Ethical Committee, and written informed consent to use the clinical and biochemical data was obtained from each participant.

The Medtronic MiniMed™ 780G system integrates a real-time continuous glucose monitor (Guardian™ Sensor 4, Medtronic MiniMed, Northridge, CA, USA), an insulin pump, and an automated insulin delivery (AID) algorithm. The system operates in two modes: a manual mode and an automatic mode (SmartGuard™ mode). In SmartGuard™ mode, the algorithm automatically adjusts basal insulin delivery every 5 min and delivers autocorrection boluses based on CGM data, aiming to maintain glucose levels close to an adjustable target (100 mg/dL, 110 mg/dL, or 120 mg/dL). Users still have to announce meals and deliver prandial boluses.

We collected device data from the pump and sensor during three time periods: baseline, 6 months, and 12 months post-automatic mode activation.

Data from the pump and sensors were downloaded from the Carelink™ platform.

At each time point, only users with at least >70% of sensor wear time were included in the analysis.

Baseline demographics, clinical characteristics, and HbA1c levels at each time period were collected. The follow-up assessments were performed according to routine clinical practice.

The primary study endpoint was the evaluation of potential sex-related differences in terms of effectiveness, safety, and impact on glucose variability of the aHCL system.

Secondary endpoints were the assessment of the overall effectiveness, safety, and impact on glucose variability of the system in a long-term period.

The main outcomes for both primary and secondary endpoints were CGM metrics (Time in Range [TIR, 70–180 mg/dL], Level 1 Time Above Range [TAR, 181–250 mg/dL], Level 2 Time Above Range [TAR2, >250 mg/dL], Level 1 Time Below Range [TBR1, 54–69 mg/dL], Level 2 Time Below Range [TBR2, <54 mg/dL]).

Secondary outcomes were glucose variability indexes (defined as reported in Supplementary Table S1) and HbA1c levels.

### Statistical Analysis

Descriptive statistics were used to summarize all results. These include mean and standard deviation (SD), minimum, maximum, and median with interquartile range (IQR) for continuous variables and counts and percentages for categorical variables. Summary statistics were reported with a maximum of 2 decimals, as appropriate.

Estimates along with their 95% Confidence Intervals (CIs) were provided.

To examine sex-related differences in TBR (divided into time in <54 mg/dL and time in 54–69 mg/dL), TIR, and TAR (divided into time in 181–250 mg/dL and time in >250 mg/dL), other CGM metrics, and HbA1c across the analysis periods, longitudinal linear models accounting for within-subject correlations were applied. Each model included time, sex, and the time-by-sex interaction as covariates and was further adjusted for baseline age and the presence of diabetes-related complications.

A subgroup analysis was included by dividing female patients into two age groups: age <45 years and age ≥45 years. This age threshold was chosen to approximate differences in reproductive status among females.

A stratified analysis was performed by dividing the cohort according to baseline HbA1c level (<7% vs. ≥7%).

A multivariable analysis adjusted for age, patient’s sex, diabetes-related complications at baseline, and HbA1c levels at baseline was performed in order to detect possible associations with a composite outcome defined as HbA1c <7.0% and TIR >70% and TBR <4% at 12 months.

All statistical tests were based on a two-sided significance level of 0.05. SAS software, version 9.4, (SAS Institute Inc., Cary, NC, USA) was used to perform statistical analyses.

## 3. Results

Overall, 176 subjects were included in the study. For each patient, only time periods with sensor usage of at least 70% were included in the analyses. In total, 173 patients met this criterion for at least one of the three analysis periods (baseline, 6 months, and 12 months). Of these, 158 contributed data for the baseline period, 137 for the 6-month period, and 135 for the 12-month period.

Mean age at baseline was 46.9 years (ranging from 20.0 to 95.0), more than half of the patients were females (65.8%), and 28.9% had diabetes-related complications at baseline. No between-sex differences in the abovementioned baseline characteristics were recorded.

Percentages of TIR, TAR, and TBR over the three time periods (baseline, 6 months, and 12 months) are reported in Table 1.

TIR significantly improved at 6 months (6.6%, 95%CI 4.5–8.6, *p* < 0.001) and at 12 months (5.4%, 95%CI 3.7–7.0, *p* < 0.001) compared to baseline.

TAR 181–250 mg/dL significantly (*p* < 0.001) decreased at 6 months (−4.5%, 95%CI −6.1 to −3.0) and at 12 months (−3.8%, 95%CI −5.0 to −2.6), compared to baseline. Similarly, there was a significant (*p*<0.001) reduction in TAR >251 mg/dL by −1.9% (−2.8 to −0.9) and −1.6% (95%CI −2.4 to −0.9) at 6 months and 12 months, respectively.

At 12 months, a significant (*p* < 0.001) improvement from baseline in blood glucose (BG) mean (−7.4, 95% CI −9.9 to −4.8), BG SD (−3.3, 95% CI −4.7 to −1.8), Glucose Management Indicator (GMI; −0.2, 95% CI −0.2 to −0.1), J index (−4.4, 95% CI −5.9 to −2.9), Continuous Overall Net Glycemic Action 4 (CONGA 4; −4.2, 95% CI −6.2 to −2.2), Mean Of Daily Differences (MODD; −4.9, 95% CI −6.5 to −3.2), Kovatchev High Blood Glucose Index (HBGI; −1.1, 95% CI −1.5 to −0.8), Blood Glucose Risk Index mean (BGRI mean; −0.6, 95%CI −0.9 to −0.3), and Average Daily Risk Range (ADRR; −2.2, 95%CI −3.3 to −1.1) was recorded. Overall, changes observed at 6 months were comparable to those at 12 months, except for BG coefficient of variation (BG CV), which resulted significantly (*p* = 0.006) reduced from baseline at 6 months (−1.3, 95% CI −2.2 to −0.4), but not at 12 months.

Mean HbA1c levels were significantly reduced from baseline at 6 months (7.5 ± 1.1% vs. 7.0 ± 0.8%; delta −0.6%, 95% CI −0.8 to −0.4%, *p* < 0.001) and the same reduction was recorded at 12 months (7.5 ± 1.1% vs. 6.9 ± 0.7%; delta −0.6%, 95%CI −0.7 to −0.4%, *p* < 0.001).

The overall cohort was stratified by the patient’s sex. The results of the models estimating sex-related differences are presented in Table 2. Most glycemic outcomes showed a statistically significant change from the baseline period at both the 6 months and 12-month periods for males. At the baseline period, a significant difference in CONGA 1 and CONGA 2 was estimated between females and males, with females exhibiting higher values (an additional 3.6 mg/dL and 3.9 mg/dL, respectively). The interaction terms in the longitudinal models were not significant for any glycemic outcome, indicating that the trajectory of change over time did not differ significantly between sexes. The only exception was HbA1c, for which females showed a significantly greater reduction from the baseline period at the 12-month period compared with males (−0.4%). TBR, TIR, TAR, CGM metrics, and HbA1c values observed across the three analysis periods for females and males are reported in Supplementary Table S2 and Supplementary Table S3, respectively.

A subgroup analysis was conducted among females to investigate age-related differences in TBR, TIR, and TAR, other CGM metrics, and HbA1c across the analysis periods, by dividing female patients into two age groups (age <45 years and age ≥45 years). No statistically significant differences emerged between age groups at baseline, and the trajectories of change over time (captured by the interaction terms of the model) did not differ significantly between groups (Table 3).

At the univariate analysis, HbA1c <7.0% at baseline was associated with the achievement of HbA1c <7.0% (OR 12.11 95%CI 2.64–55.50, *p* = 0.001), with the achievement of a TIR >70% (OR 12.35 CI 2.77–55.03, *p* < 0.001) and with the achievement of the composite outcome at 12 months (OR 2.75 95%CI 1.12–6.73, *p* = 0.027) (Table 4).

A stratified analysis was performed by dividing the cohort according to baseline HbA1c level (<7% vs. ≥7%). Supplementary Tables S4 and S5 summarize the observed TIR and HbA1c values across the three time periods, and present the mean changes estimated using longitudinal linear models that account for within-subject correlation. The tables also report the proportion of participants achieving key glycemic targets, including TIR >70%, total TBR (<70 mg/dL) <4%, and a composite target defined as TIR >70%, total TBR <4%, and HbA1c <7%.

Subjects with baseline HbA1c <7% did not exhibit a statistically significant change in HbA1c over time (mean change at 6 months: −0.2%, *p* = 0.061; mean change at 12 months: −0.1%, *p* = 0.113). Among subjects with baseline HbA1c ≥7%, a statistically significant mean reduction of 1 percentage point at both subsequent time points was detected. The estimates for mean changes in TIR within each stratum indicate a larger absolute improvement among participants with higher baseline HbA1c levels. Despite the relevant improvements in both TIR and HbA1c, only 44.4% of participants with baseline HbA1c ≥7% achieved the composite outcome at 12 months, whereas 66.7% of those with baseline HbA1c <7% met this outcome.

At the multivariable analysis adjusted for age, patient’s sex, diabetes-related complications at baseline, and HbA1c at baseline, only HbA1c <7.0% at baseline resulted in being associated with the achievement of the composite outcome (OR 2.80 95%CI 1.05–7.48, *p* = 0.04). The collinearity between predictors was investigated by calculating the Variance Inflation Factor (VIF) of each covariate. VIF was always lower than 1.5, indicating very low multicollinearity among the predictors (Table 5).

The association between baseline HbA1c level (<7% vs. ≥7%) and the odds of showing HbA1c <7% at 12 months was also investigated with a simple logistic model. Given the low variability in the outcome (only 24 subjects had HbA1c ≥7% at 12 months), no covariate adjustment was performed.

## 4. Discussion

Our data showed a significant improvement in glycemic control during treatment with aHCL in terms of HbA1c, TIR, and TAR over a one-year follow up. Mean TBR was already low at baseline and did not change significantly during the follow up.

Moreover, glycemic variability indexes, including mean glucose, CV, GMI, CONGA 4, J index, MODD, HBGI, BGRI mean, and ADRR, exhibited significant improvements over 6 and 12 months.

These results were observed consistently in both male and female participants, with a better improvement in HbA1c in females, highlighting the generalizability of the intervention across sexes.

The results of our study align with several works in the literature demonstrating the therapeutic efficacy of aHCL systems, regardless of patient’s sex. In particular, our study confirms, in a longer follow-up and on a larger population, the results obtained from another similar real-world experience. The study demonstrated an average reduction of 0.7% in HbA1c, an improvement in TIR, TAR, TBR, and several indices of glycaemic variability (SD, CV, J index, CONGA indexes, MODD, LBGI, HBGI, BGRI mean, BGRI, and ADRR) during aHCL therapy in a 6-month follow-up [6].

Furthermore, another recent study showed a significant improvement in TIR, TAR, TBR, GMI, mean glucose, and CV over a two-year period of aHCL therapy, in which patient’s sex did not appear to be correlated with increased odds of achieving a percentage of TIR >70% in both univariate and multivariate analysis [3].

These results suggest that the efficacy and safety of this aHCL system are durable and not influenced by physiological or behavioral factors related to patient’s sex.

Our results contrast with some prior literature, which pointed out differences between males and females with T1D in terms of glycaemic control, prevalence of micro- and macrovascular complications, and psychosocial aspects of disease management [7].

In fact, studies conducted in European populations of young and adult patients with T1D have shown a lower percentage of females reaching the HbA1c <7.0% target despite a higher utilization of continuous subcutaneous insulin infusion systems than males [8,9]. Although females were also more represented in our cohort—confirming a higher use of diabetes technologies—female patients in our population achieved a significantly lower HbA1c compared to males.

Conversely, registry data from patients aged 10 to 40 years reported that females tended to have higher rates of insulin pump use than males, with better glycemic control in terms of HbA1c across the observation period [10].

Similarly, a large population-based study found that male sex and social deprivation were independently associated with a lower likelihood of initiating insulin pump therapy in adult patients with T1D [11]. The absence of significant differences between the two age groups (<45 and ≥45 years old) tested among female patients in our study, used as a proxy for hormonal and reproductive status, further highlights the complexity of the mechanisms underlying both intra- and inter-sex differences, reflecting a complex interplay of psychosocial, behavioral, and systemic factors that influence diabetes care.

Furthermore, several studies have shown an increased rate of all-cause, cardiovascular, and renal mortality in female patients with T1D compared to male patients [12]. Regarding microvascular complications, albuminuric nephropathy was found to be more frequent in males, while the non-albuminuric form was more frequent in females; diabetic retinopathy and diabetic neuropathy proved to occur more frequently in men, but retinopathy-related visual impairment has been shown to occur more frequently in women [13]. In consideration of these differences, Battelino et al. [14] highlighted the importance of individualized glycemic targets, suggesting that sex-based physiological differences could influence glycemic outcomes.

In our study, in the multivariable analysis, the only variable associated with achieving HbA1c <7%, TIR >70%, and TBR <4% was having an HbA1c <7% at baseline, without the effect of sex. These findings confirm that a prior history of good glycemic control is a strong predictor of maintaining such control over time, irrespective of subsequent therapeutic changes, while the stratified analysis according to baseline HbA1c (<7.0% vs. ≥7.0%) further demonstrated that individuals with poorer glycemic control at baseline show greater clinical benefit from transitioning to aHCL therapy. It is well established that, in T1D, HbA1c alone does not fully capture the adequacy of glycemic control, as glycemic variability may differ substantially even at similar HbA1c levels.

To the best of our knowledge, our study is the first to compare a broad set of GV indexes in male and female patients with T1D on aHCL therapy. As early as 2006, GV was described as the “third component of dysglycemia”, underscoring its importance beyond mean glucose and HbA1c [15]. Several studies in the following years have shown an association between CGM-derived metrics and GV and disease complications in type 1 diabetes mellitus, especially acute complications and chronic microvascular complications, even independently of HbA1c and mean blood glucose values [16,17,18,19,20,21,22]. In particular, a study by Sartore et al. [23] highlighted a possible role of indexes such as SD, CONGA 2 and HBGI as risk factors for diabetic retinopathy in patients with T1D and T2D, while Stem et al. [19] reported that GV correlates with retinal thinning in patients with T1D, suggesting that variability in blood glucose may contribute to retinal neurodegeneration.

Overall, our findings suggest that the use of aHCL therapy may be effective in mitigating potential sex disparities, possibly due to its personalized and automated approach. The significant reductions in GV indexes reinforce prior evidence that aHCL systems can contribute in different ways to improve overall metabolic stability and to reduce the risk of disease complications, regardless of the patient’s sex.

These results highlight that once equitable access to advanced technologies is ensured, clinical benefits are comparable across sexes. This underscores the importance of addressing barriers to access to these technologies, rather than tailoring treatment algorithms by the patient’s sex.

Although the results are promising, some limitations must be acknowledged.

The analysis was conducted on a selected group of patients, and therefore, given the nature of retrospective observational research data, selection bias is possible.

The study did not evaluate potential confounding factors such as physical activity, dietary patterns, or medication adherence, which could influence glycemic outcomes, and did not evaluate long-term micro- and macrovascular complications. An additional limitation is that potential sex-related differences could be explained, at least in part, by psycho-social and behavioral factors that were not assessed in our analysis. Furthermore, although the 12-month follow-up provides valuable longitudinal information, longer-term studies are needed to determine the sustainability of these improvements. Future research should also explore the mechanistic underpinnings of sexual responses to glycemic interventions, potentially integrating biomarkers and behavioral assessments.

## 5. Conclusions

In conclusion, our findings suggest that aHCL therapy effectively improves TIR, TAR, and multiple GV indices equally in male and female patients, with a better improvement in HbA1c in female patients, participants over 6 and 12 months of follow up.

## Figures and Tables

**Table 1:** Percentage of time in range, time above range, and time below range in the study population at baseline, 6 months, and 12 months follow-up. Data are reported as mean ± SD.

	Baseline (*n* = 158)	6 Months (*n* = 137)	12 Months (*n* = 135)	Change at 6 Months vs. Baseline	Change at 12 Months vs. Baseline
Time (%) in <54 mg/dL	0.7 ± 1.1	0.6 ± 2.1	0.6 ± 1.8	0.0 (−0.3; 0.3), 0.845	0.0 (−0.2; 0.3), 0.874
Time (%) in 54–69 mg/dL	2.0 ± 1.7	1.8 ± 1.9	1.9 ± 2.4	−0.1 (−0.5; 0.2), 0.455	−0.0 (−0.3; 0.3), 0.958
Time (%) in 70–180 mg/dL	70.4 ± 13.2	76.9 ± 12.3	76.1 ± 10.7	6.6 (4.5; 8.6), <0.001	5.4 (3.7; 7.0), <0.001
Time (%) in 181–250 mg/dL	21.1 ± 8.7	16.7 ± 8.2	17.1 ± 7.6	−4.5 (−6.1; −3.0), <0.001	−3.8 (−5.0; −2.6), <0.001
Time (%) in >250 mg/dL	5.9 ± 6.9	4.0 ± 5.7	4.3 ± 4.9	−1.9 (−2.8; −0.9), <0.001	−1.6 (−2.4; −0.9), <0.001

**Table 2:** Effect of sex on glycemic outcomes across analysis periods. Estimated changes are expressed as mean change, 95% CI, and *p*-value. Estimates are adjusted for baseline age and the presence of diabetes-related complications. (BG: blood glucose; SD: standard deviation; CV: coefficient of variation; GMI: glucose management index; CONGA: continuous overall net glycemia action; MODD: mean of daily differences; LBGI: low blood glucose index; HBGI: high blood glucose index; BGRI: Blood Glucose Risk Index; ADRR: average daily risk range.

	Main Effects			Interaction Terms	
	Change at 6 Months vs. Baseline (Male)	Change at 12 Months vs. Baseline (Male)	Female vs. Male (at Baseline)	Extra Change for Females at 6 Months	Extra Change for Females at 12 Months
Time (%) in <54 mg/dL	−0.1 (−0.6; 0.4), 0.717	0.0 (−0.4; 0.5), 0.828	0.1 (−0.5; 0.6), 0.756	0.1 (−0.5; 0.7), 0.771	0.0 (−0.6; 0.5), 0.871
Time (%) in 54–69 mg/dL	−0.2 (−0.9; 0.4), 0.482	−0.0 (−0.6; 0.5), 0.915	0.1 (−0.6; 0.7), 0.871	0.1 (−0.7; 1.0), 0.755	0.0 (−0.7; 0.7), 0.920
Time (%) in 70–180 mg/dL	7.6 (4.0; 11.2), <0.001	6.2 (3.4; 9.0), <0.001	0.3 (−3.7; 4.3), 0.895	−1.5 (−5.9; 2.9), 0.509	−1.1 (−4.6; 2.3), 0.515
Time (%) in 181–250 mg/dL	−4.9 (−7.6; −2.3), <0.001	−3.9 (−6.1; −1.8), <0.001	0.2 (−2.5; 2.9), 0.864	0.6 (−2.7; 3.9), 0.714	0.1 (−2.5; 2.8), 0.922
Time (%) in >250 mg/dL	−2.4 (−4.0; −0.7), 0.005	−2.3 (−3.6; −1.0), <0.001	−0.7 (−2.6; 1.3), 0.502	0.7 (−1.3; 2.7), 0.506	1.0 (−0.5; 2.6), 0.200
SG Mean (mg/dL)	−8.9 (−14.6; −3.2), 0.002	−7.4 (−11.8; −3.0), 0.001	−0.3 (−6.6; 6.1), 0.937	0.9 (−6.0; 7.9), 0.790	0.2 (−5.2; 5.7), 0.934
SG SD (mg/dL)	−6.0 (−9.1; −2.9), <0.001	−4.4 (−6.8; −1.9), <0.001	0.5 (−2.9; 3.9), 0.779	1.9 (−2.0; 5.7), 0.343	1.8 (−1.2; 4.8), 0.236
SG CV (%)	−2.0 (−3.7; −0.4), 0.017	−1.3 (−2.6; 0.1), 0.060	0.6 (−1.2; 2.3), 0.521	0.9 (−1.1; 3.0), 0.366	1.0 (−0.6; 2.6), 0.234
GMI (%)	−0.2 (−0.3; −0.1), 0.002	−0.2 (−0.3; −0.1), 0.001	−0.0 (−0.2; 0.1), 0.937	0.0 (−0.1; 0.2), 0.790	0.0 (−0.1; 0.1), 0.934
J index (mg/dL^2^)	−6.1 (−9.4; −2.8), <0.001	−4.9 (−7.4; −2.4), <0.001	−0.2 (−4.1; 3.6), 0.899	1.2 (−2.8; 5.2), 0.551	0.9 (−2.2; 4.0), 0.576
CONGA 1 (mg/dL)	−0.8 (−3.2; 1.5), 0.474	−0.6 (−2.5; 1.2), 0.486	3.6 (1.2; 6.1), 0.004	−0.7 (−3.5; 2.1), 0.618	1.0 (−1.2; 3.2), 0.376
CONGA 2 (mg/dL)	−3.2 (−6.7; 0.2), 0.062	−2.9 (−5.6; −0.2), 0.036	3.9 (0.3; 7.5), 0.034	−0.6 (−4.8; 3.6), 0.781	2.0 (−1.3; 5.3), 0.229
CONGA 4 (mg/dL)	−6.8 (−11.0; −2.5), 0.002	−5.7 (−9.1; −2.4), <0.001	1.8 (−2.6; 6.3), 0.415	0.3 (−4.8; 5.5), 0.898	2.8 (−1.3; 6.9), 0.182
MODD (mg/dL)	−5.9 (−9.5; −2.4), 0.001	−5.7 (−8.6; −2.9), <0.001	1.2 (−2.3; 4.8), 0.502	−0.3 (−4.6; 4.0), 0.895	1.2 (−2.2; 4.7), 0.486
Kovatchev LBGI	−0.1 (−0.3; 0.2), 0.652	−0.0 (−0.2; 0.2), 0.972	−0.0 (−0.2; 0.2), 0.968	0.1 (−0.2; 0.4), 0.640	0.0 (−0.2; 0.3), 0.729
Kovatchev HBGI	−1.5 (−2.3; −0.7), <0.001	−1.3 (−1.9; −0.6), <0.001	−0.1 (−1.1; 0.8), 0.794	0.3 (−0.7; 1.3), 0.596	0.2 (−0.6; 1.0), 0.599
BGRI mean	−1.5 (−2.3; −0.8), <0.001	−1.3 (−1.8; −0.7), <0.001	−0.1 (−1.0; 0.8), 0.787	0.3 (−0.6; 1.3), 0.477	0.2 (−0.5; 1.0), 0.502
BGRI SD	−1.2 (−1.9; −0.5), 0.001	−0.7 (−1.3; −0.2), 0.009	0.2 (−0.6; 1.0), 0.662	0.4 (−0.5; 1.2), 0.414	0.2 (−0.4; 0.9), 0.499
ADRR	−3.4 (−5.8; −1.0), 0.005	−2.9 (−4.7; −1.0), 0.002	2.3 (−0.3; 4.9), 0.077	0.9 (−2.0; 3.8), 0.555	1.3 (−0.9; 3.6), 0.249
HbA1c (%)	−0.6 (−0.9; −0.2), 0.002	−0.4 (−0.7; −0.1), 0.022	0.3 (−0.1; 0.6), 0.115	0.0 (−0.4; 0.5), 0.861	−0.4 (−0.8; −0.0), 0.049

**Table 3:** Effect of age on glycemic outcomes across analysis periods in females. Estimated changes are expressed as mean change, 95% CI, and *p*-value. Estimates are adjusted for the baseline presence of diabetes-related complications.

	Main Effects			Interaction Terms	
	Change at 6 Months vs. Baseline. (Age < 45)	Change at 12 Months vs. Baseline. (Age < 45)	Age ≥ 45 vs. Age < 45 (at Baseline)	Extra Change for Subjects with Age ≥ 45 at 6 Months	Extra Change for Subjects with Age ≥ 45 at 12 Months
Time (%) in <54 mg/dL	0.1 (−0.4; 0.6), 0.793	0.1 (−0.3; 0.5), 0.702	−0.3 (−1.1; 0.4), 0.361	−0.1 (−0.8; 0.6), 0.736	−0.1 (−0.7; 0.4), 0.629
Time (%) in 54–69 mg/dL	−0.1 (−0.9; 0.6), 0.714	0.3 (−0.3; 0.9), 0.335	−0.1 (−0.9; 0.7), 0.852	0.0 (−1.0; 1.0), 0.937	−0.6 (−1.4; 0.3), 0.186
Time (%) in 70–180 mg/dL	4.8 (1.2; 8.4), 0.009	4.4 (1.5; 7.3), 0.003	−1.4 (−6.1; 3.3), 0.547	2.4 (−2.6; 7.4), 0.343	1.2 (−2.8; 5.2), 0.552
Time (%) in 181–250 mg/dL	−3.6 (−6.3; −1.0), 0.008	−3.9 (−6.1; −1.7), <0.001	1.1 (−2.1; 4.3), 0.502	−1.2 (−4.9; 2.5), 0.507	0.2 (−2.8; 3.3), 0.877
Time (%) in >250 mg/dL	−1.1 (−2.8; 0.6), 0.203	−0.9 (−2.2; 0.4), 0.185	0.7 (−1.6; 3.1), 0.539	−1.1 (−3.4; 1.3), 0.366	−0.7 (−2.6; 1.2), 0.457
SG Mean (mg/dL)	−6.0 (−11.9; −0.1), 0.048	−7.1 (−11.8; −2.3), 0.004	3.4 (−4.2; 11.0), 0.381	−3.5 (−11.7; 4.7), 0.398	−0.1 (−6.7; 6.5), 0.975
SG SD (mg/dL)	−2.3 (−5.4; 0.7), 0.134	−1.8 (−4.2; 0.7), 0.157	3.6 (−0.6; 7.7), 0.090	−3.4 (−7.6; 0.8), 0.116	−1.4 (−4.8; 2.0), 0.411
SG CV (%)	−0.6 (−2.2; 1.1), 0.492	0.0 (−1.3; 1.4), 0.977	1.4 (−0.7; 3.5), 0.201	−1.0 (−3.3; 1.3), 0.396	−0.5 (−2.4; 1.3), 0.581
GMI (%)	−0.1 (−0.3; −0.0), 0.048	−0.2 (−0.3; −0.1), 0.004	0.1 (−0.1; 0.3), 0.381	−0.1 (−0.3; 0.1), 0.398	−0.0 (−0.2; 0.2), 0.975
J index (mg/dL^2^)	−3.3 (−6.7; 0.0), 0.052	−3.5 (−6.2; −0.8), 0.010	2.5 (−2.1; 7.1), 0.292	−2.9 (−7.5; 1.8), 0.221	−0.9 (−4.6; 2.8), 0.617
CONGA 1 (mg/dL)	−1.1 (−3.6; 1.3), 0.363	−0.4 (−2.3; 1.6), 0.715	0.6 (−2.6; 3.8), 0.705	−0.8 (−4.1; 2.6), 0.654	1.5 (−1.2; 4.2), 0.286
CONGA 2 (mg/dL)	−3.0 (−6.5; 0.6), 0.101	−1.3 (−4.2; 1.5), 0.366	1.8 (−2.7; 6.4), 0.423	−1.6 (−6.5; 3.3), 0.517	1.0 (−3.0; 4.9), 0.625
CONGA 4 (mg/dL)	−5.1 (−9.3; −0.8), 0.021	−2.7 (−6.1; 0.8), 0.129	3.6 (−1.9; 9.1), 0.193	−2.6 (−8.5; 3.4), 0.394	−0.4 (−5.2; 4.4), 0.872
MODD (mg/dL)	−4.3 (−7.7; −0.8), 0.016	−3.9 (−6.7; −1.1), 0.006	3.0 (−1.3; 7.4), 0.168	−3.7 (−8.4; 1.1), 0.129	−1.0 (−4.9; 2.9), 0.605
Kovatchev LBGI	0.0 (−0.2; 0.3), 0.933	0.1 (−0.1; 0.3), 0.252	−0.1 (−0.4; 0.2), 0.448	0.0 (−0.3; 0.4), 0.953	−0.2 (−0.4; 0.1), 0.290
Kovatchev HBGI	−0.9 (−1.8; −0.1), 0.038	−0.9 (−1.6; −0.3), 0.007	0.4 (−0.7; 1.6), 0.468	−0.6 (−1.7; 0.6), 0.347	−0.2 (−1.1; 0.7), 0.688
BGRI mean	−0.9 (−1.7; −0.1), 0.028	−0.8 (−1.5; −0.2), 0.011	0.3 (−0.8; 1.4), 0.589	−0.5 (−1.6; 0.6), 0.325	−0.3 (−1.2; 0.5), 0.443
BGRI SD	−0.4 (−1.1; 0.3), 0.244	−0.3 (−0.8; 0.2), 0.275	0.7 (−0.2; 1.7), 0.117	−0.8 (−1.7; 0.2), 0.105	−0.4 (−1.1; 0.4), 0.335
ADRR	−1.7 (−4.2; 0.8), 0.173	−0.8 (−2.8; 1.1), 0.397	2.4 (−0.9; 5.7), 0.156	−1.5 (−4.9; 1.9), 0.387	−1.3 (−4.0; 1.5), 0.364
HbA1c (%)	−0.6 (−1.0; −0.2), 0.004	−0.9 (−1.2; −0.5), <0.001	−0.0 (−0.4; 0.3), 0.861	0.1 (−0.4; 0.6), 0.672	0.2 (−0.2; 0.7), 0.292

**Table 4:** Univariate logistic regression investigating the association of baseline age, sex, presence of diabetes-related complications, and HbA1c level (<7% vs. ≥7%) with the odds of meeting HbA1c <7%, TIR >70%, TBR <4% or the composite outcome (HbA1c < 7%, TIR > 70% and TBR < 4%) at 12 months.

Outcome	Parameter	Odds Ratio (OR) (95% CI)	*p*-Value
HbA1c < 7%	Age	0.99 (0.96–1.03)	0.717
	Sex (Female)	1.20 (0.46–3.11)	0.703
	HbA1c at baseline <7%	12.11 (2.64–55.50)	0.001
	Complications at baseline	0.67 (0.27–1.66)	0.386
TIR > 70%	Age	1.01 (0.98–1.04)	0.596
	Sex (Female)	1.34 (0.62–2.93)	0.458
	HbA1c at baseline <7%	12.35 (2.77–55.03)	<0.001
	Complications at baseline	1.13 (0.49–2.64)	0.769
TBR < 4%	Age	1.02 (0.98–1.06)	0.290
	Sex (Female)	1.59 (0.61–4.17)	0.342
	HbA1c at baseline <7%	0.42 (0.15–1.17)	0.098
	Complications at baseline	2.58 (0.71–9.37)	0.149
Composite outcome	Age	1.01 (0.98–1.04)	0.542
	Sex (Female)	0.95 (0.39–2.32)	0.903
	HbA1c at baseline <7%	2.75 (1.12–6.73)	0.027
	Complications at baseline	0.96 (0.40–2.29)	0.928

**Table 5:** Multivariable logistic regression investigating the association of baseline age, sex, presence of diabetes-related complications, and HbA1c level (<7% vs. ≥7%) with the odds of meeting the composite outcome at 12 months.

Parameter	Odds Ratio (OR) (95% CI)	*p*-Value
Age	1.02 (0.98–1.06)	0.363
Sex (Female)	1.15 (0.42–3.15)	0.793
HbA1c at baseline <7%	2.80 (1.05–7.48)	0.040
Complications at baseline	0.92 (0.30–2.79)	0.883

No severe hypoglycemia, diabetic ketoacidosis (DKA), or hospitalization for diabetes-related causes occurred during the follow up, in both sexes.

## Data Availability

The data presented in this study are available on request from the corresponding author.

## Supplementary Materials

The following supporting information can be downloaded at: https://www.mdpi.com/article/10.3390/jcm14248823/s1, Table S1. Glycemic variability indexes and their definitions. (BG: blood glucose; SD: standard deviation; CV: coefficient of variation; GMI: glucose management index; CONGA: continuous overall net glycemia action; MODD: mean of daily differences; LBGI: low blood glucose index; HBGI: high blood glucose index; BGRI: Blood Glucose Risk Index; ADRR: average daily risk range); Table S2. Glycemic Outcomes in females. Data are reported as mean ± SD; Table S3. Glycemic Outcomes in males. Data are reported as mean ± SD; Table S4. Change in TIR and HbA1c in subjects with HbA1c <7% at baseline. Estimated changes are expressed as mean change, 95% CI, and *p*-value; Table S5. Change in TIR and HbA1c in subjects with HbA1c ≥7% at baseline. Estimated changes are expressed as mean change, 95% CI, and *p*-value.
