# Supplementary material for: Sex Differences in Continuous Glucose Monitoring Metrics and Glucose Variability in Subjects with Type 1 Diabetes Treated with Advanced Hybrid Closed Loop Therapy: An Observational, Retrospective, One-Year Follow-Up Study
Source: J Clin Med. 2025 Dec 13;14(24):8823. doi: 10.3390/jcm14248823 (PMC12734264; doi:10.3390/jcm14248823)
Supplement: Supplementary file 1 [file jcm-14-08823-s001.zip › Table S1.pdf]

**Supplementary Table S1.** Glycemic variability indexes and their definitions. (BG: blood glucose; SD: standard deviation; CV: coefficient of variation; GMI: glucose management index; CONGA: continuous overall net glycemia action; MODD: mean of daily differences; LBGI: low blood glucose index; HBGI: high blood glucose index; BGRI: Blood Glucose Risk Index; ADRR: average daily risk range).

| Variable | Derivation |
|---|---|
| **Number of days with sensor active** | Number of days with at least one CGM measurement |
| **Sensor usage (%)** | Number of CGM measurements / (Number of days in the period of interest * 288) * 100 |
| **BG Mean, SD, and CV** | Mean, SD, and CV of CGM measurements |
| **GMI** | 3.31 + (0.02392 * BG Mean) |
| **J index** | 0.001 * ((BG Mean + BG SD) ^ 2) |
| **CONGA-X** | CONGA-X is computed by taking the SD of differences in CGM measurements separated by X hours |
| **MODD** | MODD is calculated as the mean of absolute differences between CGM measurements taken at the same time point on consecutive days |
| **HBGI** | HBGI is calculated as the mean of (10 ∗ fbg ^ 2), where for each CGM measurement fbg is given by max (0, 1.509 * (log (SG) ^ (1.084) – 5.381)) |
| **LBGI** | LBGI is calculated as the mean of (10 ∗ fbg ^ 2), where for each CGM measurement fbg is given by min (0, 1.509 * (log (SG) ^ (1.084) – 5.381)) |
| **BGRI Mean** | BGRI Mean is computed as the average of (10 ∗ fbg ^ 2), where for each CGM measurement fbg is given by 1.509 * (log (SG) ^ (1.084) – 5.381) |
| **BGRI SD** | BGRI SD is computed as the standard deviation of (10 ∗ fbg ^ 2), where for each CGM measurement fbg is given by 1.509 * (log (SG) ^ (1.084) – 5.381) |
| **ADRR** | For each day of the period of interest, the maximum values of the following two measures are calculated and added together:<br>• (10 ∗ fbg ^ 2), where for each CGM measurement fbg is given by max (0, 1.509 * (log (SG) ^ (1.084) – 5.381))<br>• (10 ∗ fbg ^ 2), where for each CGM measurement fbg is given by min (0, 1.509 * (log (SG) ^ (1.084) – 5.381))<br>ADRR is then calculated as the mean of the daily values thus obtained. |
