# Supplementary material for: Sex Differences in Continuous Glucose Monitoring Metrics and Glucose Variability in Subjects with Type 1 Diabetes Treated with Advanced Hybrid Closed Loop Therapy: An Observational, Retrospective, One-Year Follow-Up Study
Source: J Clin Med. 2025 Dec 13;14(24):8823. doi: 10.3390/jcm14248823 (PMC12734264; doi:10.3390/jcm14248823)
Supplement: Supplementary file 1 [file jcm-14-08823-s001.zip › Table S2.pdf]

Supplementary Table S2. Glycemic Outcomes in females. Data are reported as mean ± SD.

	Baseline (N = 105)	6 months (N = 92)	12 months (N = 87)
**Time (%) in <54 mg/dL**	0.7 ± 1.2	0.7 ± 2.4	0.6 ± 1.6
**Time (%) in 54-69 mg/dL**	2.0 ± 1.7	1.9 ± 1.9	1.9 ± 2.4
**Time (%) in 70-180 mg/dL**	70.5 ± 12.1	76.4 ± 11.7	75.7 ± 10.1
**Time (%) in 181-250 mg/dL**	21.2 ± 8.0	17.0 ± 8.1	17.2 ± 7.2
Time (%) in >250 mg/dL	5.6 ± 6.6	4.0 ± 5.2	4.5 ± 4.6
SG Mean (mg/dL)	152.6 ± 19.8	145.0 ± 18.6	145.7 ± 17.4
SG SD (mg/dL)	51.3 ± 9.8	47.1 ± 10.4	48.4 ± 10.7
SG CV (%)	33.6 ± 4.9	32.5 ± 5.5	33.1 ± 5.2
GMI (%)	7.0 ± 0.5	6.8 ± 0.4	6.8 ± 0.4
J index (mg/dL^2)	42.3 ± 12.3	37.6 ± 10.8	38.4 ± 10.4
CONGA 1 (mg/dL)	42.5 ± 7.6	40.7 ± 7.8	42.2 ± 8.0
CONGA 2 (mg/dL)	60.2 ± 10.7	56.1 ± 10.9	58.5 ± 11.6
CONGA 4 (mg/dL)	71.6 ± 13.3	64.9 ± 13.1	68.2 ± 14.2
MODD (mg/dL)	50.7 ± 10.4	44.4 ± 11.0	45.7 ± 10.3
Kovatchev LBGI	0.8 ± 0.6	0.8 ± 0.9	0.8 ± 0.8
Kovatchev HBGI	5.9 ± 3.1	4.7 ± 2.7	4.9 ± 2.4
BGRI mean	6.6 ± 3.0	5.5 ± 2.5	5.6 ± 2.2
BGRI SD	7.6 ± 2.3	6.9 ± 2.3	7.1 ± 2.3
ADRR	36.3 ± 7.7	33.5 ± 8.1	34.3 ± 8.3
HbA1c (%)	7.6 ± 1.0	7.1 ± 0.8	6.9 ± 0.6
