# Supplementary material for: Sex Differences in Continuous Glucose Monitoring Metrics and Glucose Variability in Subjects with Type 1 Diabetes Treated with Advanced Hybrid Closed Loop Therapy: An Observational, Retrospective, One-Year Follow-Up Study
Source: J Clin Med. 2025 Dec 13;14(24):8823. doi: 10.3390/jcm14248823 (PMC12734264; doi:10.3390/jcm14248823)
Supplement: Supplementary file 1 [file jcm-14-08823-s001.zip › Table S3.pdf]

Supplementary Table S3. Glycemic Outcomes in males. Data are reported as mean ± SD.

	Baseline (N = 53)	6 months (N = 45)	12 months (N = 48)
Time (%) in <54 mg/dL	0.6 ± 0.9	0.5 ± 1.1	0.6 ± 2.1
Time (%) in 54-69 mg/dL	1.9 ± 1.6	1.6 ± 2.0	1.9 ± 2.4
Time (%) in 70-180 mg/dL	70.1 ± 15.4	77.9 ± 13.5	76.8 ± 11.7
Time (%) in 181-250 mg/dL	21.0 ± 9.9	16.0 ± 8.3	16.9 ± 8.3
Time (%) in >250 mg/dL	6.3 ± 7.6	4.0 ± 6.5	3.9 ± 5.4
SG Mean (mg/dL)	153.1 ± 23.2	144.1 ± 20.6	145.0 ± 19.4
SG SD (mg/dL)	50.6 ± 12.3	44.6 ± 10.6	46.2 ± 11.1
SG CV (%)	32.9 ± 5.2	30.9 ± 5.7	31.8 ± 5.9
GMI (%)	7.0 ± 0.6	6.8 ± 0.5	6.8 ± 0.5
J index (mg/dL^2)	42.6 ± 14.4	36.4 ± 11.8	37.3 ± 11.6
CONGA 1 (mg/dL)	38.2 ± 8.4	36.9 ± 8.1	37.0 ± 7.4
CONGA 2 (mg/dL)	55.5 ± 12.1	51.6 ± 11.5	51.9 ± 10.4
CONGA 4 (mg/dL)	69.4 ± 15.4	62.1 ± 14.1	63.1 ± 13.3
MODD (mg/dL)	49.5 ± 12.5	43.5 ± 11.3	43.5 ± 10.2
Kovatchev LBGI	0.7 ± 0.5	0.7 ± 0.6	0.7 ± 0.9
Kovatchev HBGI	6.0 ± 3.6	4.5 ± 3.2	4.7 ± 2.8
BGRI mean	6.8 ± 3.4	5.2 ± 3.0	5.4 ± 2.7
BGRI SD	7.5 ± 2.9	6.3 ± 2.4	6.7 ± 2.5
ADRR	33.8 ± 8.2	30.0 ± 8.4	30.5 ± 7.5
HbA1c (%)	7.3 ± 1.3	6.8 ± 0.6	7.0 ± 0.7
