# Supplementary material for: Sex Differences in Continuous Glucose Monitoring Metrics and Glucose Variability in Subjects with Type 1 Diabetes Treated with Advanced Hybrid Closed Loop Therapy: An Observational, Retrospective, One-Year Follow-Up Study
Source: J Clin Med. 2025 Dec 13;14(24):8823. doi: 10.3390/jcm14248823 (PMC12734264; doi:10.3390/jcm14248823)
Supplement: Supplementary file 1 [file jcm-14-08823-s001.zip › Table S4.pdf]

Supplementary Table S4. Change in TIR and HbA1c in subjects with HbA1c < 7% at baseline. Estimated changes are expressed as mean change, 95% CI, and p-value.

	Summary statistics	Baseline (N = 53)	6 months (N = 37)	12 months (N = 41)	Change at 6 mont hs vs bsl.	Change at 12 mont hs vs bsl.
**Time (%) in 70-180 mg/dL**	Mean ± SD	78.7 ± 7.7	83.1 ± 7.3	81.8 ± 7.8	3.9 (1.3; 6.6), **0.004**	2.4 (0.3; 4.4), **0.024**
**HbA1c (%)**	Mean ± SD	6.6 ± 0.4	6.4 ± 0.5	6.5 ± 0.5	-0.2 (-0.4; 0.0), 0.061	-0.1 (-0.2; 0.0), 0.113
**HbA1c**						
**< 7%**	% (n/Available Measures)	100.0% (53/53)	95.8% (23/24)	93.3% (28/30)	-	-
**7-9%**	% (n/Available Measures)	0.0% (0/53)	4.2% (1/24)	6.7% (2/30)		
**> 9%**	% (n/Available Measures)	0.0% (0/53)	0.0% (0/24)	0.0% (0/30)		
**Time in 70-180 mg/dL > 70%**	% (n/Available Measures)	81.1% (43/53)	94.6% (35/37)	95.1% (39/41)	-	-
**Time in <70 mg/dL < 4%**	% (n/Available Measures)	69.8% (37/53)	83.8% (31/37)	78.0% (32/41)	-	-
**HbA1c < 7% and Time in 70-180 mg/dL > 70% and Time in <70 mg/dL < 4%**	% (n/Available Measures)	60.4% (32/53)	62.5% (15/24)	66.7% (20/30)	-	-
