# Supplementary material for: Sex Differences in Continuous Glucose Monitoring Metrics and Glucose Variability in Subjects with Type 1 Diabetes Treated with Advanced Hybrid Closed Loop Therapy: An Observational, Retrospective, One-Year Follow-Up Study
Source: J Clin Med. 2025 Dec 13;14(24):8823. doi: 10.3390/jcm14248823 (PMC12734264; doi:10.3390/jcm14248823)
Supplement: Supplementary file 1 [file jcm-14-08823-s001.zip › Table S5.pdf]

Supplementary Table S5. Change in TIR and HbA1c in subjects with HbA1c ≥7% at baseline. Estimated changes are expressed as mean change, 95% CI, and p-value.

	Summary statistics	Baseline (N = 88)	6 months (N = 74)	12 months (N = 67)	Change at 6 mont hs vs bsl.	Change at 12 mont hs vs bsl.
**Time (%) in 70-180 mg/dL**	Mean ± SD	66.2 ± 12.1	77.1 ± 9.0	75.3 ± 8.9	10.6 (7.8; 13.3), <0.001	8.1 (5.7; 10.4), <0.001
**HbA1c (%)**	Mean ± SD	8.0 ± 1.1	7.1 ± 0.5	7.0 ± 0.6	-1.0 (-1.2; -0.7), <0.001	-1.0 (-1.3; -0.8), <0.001
**HbA1c**						
**< 7%**	% (n/Available Measures)	0.0% (0/88)	44.4% (24/54)	59.3% (32/54)	-	-
**7-9%**	% (n/Available Measures)	87.5% (77/88)	55.6% (30/54)	40.7% (22/54)		
**> 9%**	% (n/Available Measures)	12.5% (11/88)	0.0% (0/54)	0.0% (0/54)		
**Time in 70-180 mg/dL > 70%**	% (n/Available Measures)	39.8% (35/88)	78.4% (58/74)	68.7% (46/67)	-	-
**Time in <70 mg/dL < 4%**	% (n/Available Measures)	87.5% (77/88)	89.2% (66/74)	91.0% (61/67)	-	-
**HbA1c < 7% and Time in 70-180 mg/dL > 70% and Time in <70 mg/dL < 4%**	% (n/Available Measures)	0.0% (0/88)	33.3% (18/54)	44.4% (24/54)	-	-
